# Reported corporate misconducts: The impact on the financial markets

**DOI:** 10.1371/journal.pone.0276637

**Published:** 2023-02-09

**Authors:** Riste Ichev

**Affiliations:** Faculty of Economics, University of Ljubljana, Ljubljana, Slovenia; BeiHang University School of Economics and Management, CHINA

## Abstract

This study empirically examines how reported corporate misconducts affect the stock returns of US firms. As the reported misconducts are broadcasted in the newspaper outlets, the cumulative abnormal return (CAR) is -4.1%. Involvement in a reported corporate misconduct gets punished by market participants especially when the act of reported misconduct is blamed on the level of the corporation rather than in involvement of a specific individual, when reported misconducts take place in the home market, and when the linguistic tone used in the newspaper article is negative. Financial penalties imposed, firm size, leverage, revenue growth, and the level of firm foreign exposure are found to have significant impact on the returns during the period of observation. The results suggest that investors recognize the importance to penalize firms in the financial markets when firms act unethically.

## Introduction

Recent reported corporate misconducts at prominent firms such as Wells Fargo, Toyota Motor Company, and Volkswagen appear to have shaken the confidence of the market participants worldwide. On 24^th^ of February 2020, Wells Fargo, the fourth largest bank in the US, acknowledged to pay 3 billion dollars to settle its long-running civil and criminal probes of its rampant fraudulent sales practices (Kelly, 2020) [1]. Within similar lines, the US Environmental Protection Agency accused Toyota Motor Company and Volkswagen to a multilayer violation of the Clean Air Act for installing a software in their vehicles designed to cheat on emissions tests (Tabuchi, 2021) [2]. The announcement of these reported corporate misconducts triggered intense and extensive media coverage, which brought these firms under heavy criticism by the stakeholders. In the case of Volkswagen, the stock value dropped about 50% only few days after the initial media announcement (La Monica, 2015) [3]. After publicly revealing the existence of a reported corporate misconduct, a drop in firms’ stock price is not surprising. A reason behind is that not only will the reported corporate misconduct impose direct costs towards the firms in the form of civil and other legal penalties, and recalls, but it will additionally alter how market participants, consumers, suppliers, and the broader public see these firms in future (Carberry et al., 2018) [4].

Along these lines, this study quantifies the current estimate of the economic impact of the reported corporate misconducts upon the stocks listed on NYSE and NASDAQ Composite, in the period from January 1995 to January 2020. It observes the economic impact of the reported corporate misconducts by their type, by the management level within the firm that conducted the reported misconduct, the location of the reported misconduct, and the linguistic tone of the newspaper article through which the act of reported misconduct is disseminated to the public. This study finds that the overall cumulative abnormal returns (CARs) are negative prior, and up to 5 days after the first reported corporate misconduct case in the media. The magnitude of the negative CARs differs across multiple types of reported misconduct, from which the *environmental violation* reported misconducts yield the largest negative coefficient–on average -9.2% for the period of up to five days after the initial announcement. Observing the initiator of the reported corporate misconduct, the findings indicate that blaming the act of a reported corporate misconduct on the level of *corporation* rather than pointing at a specific *C-level* individual hurts firm value more. Other findings show that reported corporate misconducts occurring in the *home* market more negatively affect firms’ stock returns, as well as it does the *negative* linguistic tone used to write the newspaper articles, and the information about the financial *penalty* assigned to each firm conducting the specific reported corporate misconduct. Lastly, regression analyses show that firm *size*, *leverage*, *revenue growth*, and the level of firm *foreign exposure* have significant impact on the returns when analyzing firm financial characteristics when reported corporate misconducts occur.

As it appears, some previous studies observing reported corporate misconducts and media coverage around the reported corporate misconducts are well known in the literature (for e.g. Carberry et al., 2018 [4]; Engelen, 2009 [5]; Karpoff et al, 2008 [6]; and other). However, the immediate relation between stock returns, the type and level of the reported misconducts, the geographic proximity of the reported corporate misconducts to the financial markets, and the linguistic tone of the articles disseminating the information for the act of reported misconduct, have not yet been precisely addressed in the previous literature. This study contributes to the literature by examining the effect of investor sentiment on the financial markets by quantifying four confounding effects of the reported corporate misconducts. First and foremost, the types of reported misconducts are unequally perceived by the market participants. Second, firms spin off and divert signaling information to temper investor reactions when reported corporate misconducts are blamed on the corporation rather than to specific individuals from the management. Third, market participants react more to the events that are within shorter geographic proximity to them, and lastly, the net negative linguistic tone used in the newspaper articles significantly contributes to the decrease of the market returns.

The remainder of the paper is structured as follows. Section 2 provides a theoretical background. Section 3 describes the data. Section 4 presents the methods used. Section 5 presents the results, and section 6 concludes.

## Theoretical background

The main hypothesis developing the plot of this study is nested in the observed relations between: i) companies’ exposure to the reported corporate misconducts in the media, ii) companies’ exposure to the reported misconducts taking place in different geographic regions, iii) the type and level of the reported corporate misconducts, and iv) the sentiment surrounding the linguistic tone of the articles through which the information about the reported misconduct is broadcasted to the public.

Several studies observe the relationship between investor sentiment and asset pricing (De Long et al., 1990 [7]; Kaplanski and Levy, 2010a, b [8, 9]; Cen & Liyan-Yang, 2013 [10]; Donadelli, 2016b [11]). Within these lines, this paper is related to Kapalnski & Levy (2010a, b) [8, 9] who study the impact of negative events, such as aviation disasters, on investor sentiment, and stock prices. They show evidence that aviation disasters negatively affect investor sentiment and increase fear for trading up to few days after the event. On the contrary, sporting events associated with pleasure and good mood positively affect investor sentiment, and thus stock returns.

Another set of studies to which this paper relates to examine the relationship between the media as an information disseminator and firms’ stock returns. Klibanoff et al. (1998) [12] find that investors praise more the news to which more attention has been given by the media than the news to which less attention has been assigned regardless of the news bearing same fundamental value. Blendon et al. (2004) [13] examine the intensity of media coverage of disease outbreaks. They find that the media tends to disproportionately cover dramatic events—the ones that affect many people at once. Peress (2014) [14] observes the impact of media coverage on trading and price formation by observing newspaper strikes. He shows that, on strike days, return volatility is reduced by 7%, aggregate returns show no statistically significant signals, and trading volume falls by 12%.

Francis et al. (2007) [15] and Engelberg & Parsons (2011) [16] study the role of geographic location on investor behavior and companies’ stock performance. Both studies find that geographic proximity affects the dissemination of information and thus the firms’ stock market performance. In addition, they find that rural firms exhibit higher costs of debt than the firms located in urban areas.

Another close relation of this study is to Carberry et al. (2018) [4] who study the stock market reaction to unethical corporate behavior. They argue that signaling effects coming from the information about a specific reported misconduct are important for investors since firms are strongly inclined to limit the information they disclose about the reported misconduct. In addition, they record a negative stock market reaction to the unethical corporate behavior.

Last but not least, this study also relates to the literature examining linguistic tone of media communication. Within this literature strain, Chen et al. (2016) [17] and Wagner et al. (2018) [18] examine content of political speeches and find that net positive linguistic tone increases market returns and trading volume, and on the contrary, negative linguistic tone has the opposite effect. In addition, Chen et al. (2016) [17] conclude that stock market participants are quite sensitive to news disseminated by powerful people, such as politicians, since even politicians that may not yet be in power, can influence the stock market and its performance.

## Data

This study considers a comprehensive set of 1,269 newspaper published articles on reported corporate misconduct actions undertaken by 462 (S&P500, NASDAQ Composite, and NYSE Composite) listed firms in the period from January 1995 to January 2020. To make sure that the article source is reliable and reaching broad masses, the three largest by circulation US newspapers—“The New York Times, “The Wall Street Journal”, and “The USA Today” have been taken into account. The reported acts of corporate misconduct are identified via the ProQuest article search engine using Carberry et al. (2018)’s [4] set of keywords in English: *financial misrepresentation*, *accounting fraud and tax fraud*, *forgery*, *swindling*, *corruption and bribery*, *insider trading*, *insider dealing*, *cartel*, *price fixing and market abuse*, *anti-trust*, *monopoly*, *human rights and discrimination*, *environmental violation*, *environmental damage*, *and pollution*. More specifically, about 80% of the articles collected come from “The New York Times”, and the rest is from “The Wall Street Journal” and “The USA Today” combined. As noted by Berlinger et al. (2022) [19], it is important to stress that not all corporate misconducts become public. Journalists can be more concentrated on large scandals, specific industries, and specific types of misconducts. However, in this study at least to some extent, the potential missing reporting bias diminishes as the focus is directed to only the *reported* corporate misconducts as it is assumed that all investors are equally informed.

The set of newspaper articles is then manually categorized according to whether the reported misconduct was committed on the level of the *corporation* or the *C-level (CEO*, *CFO*, *board member)*, whether firms committed the reported corporate misconducts in the *home (in The US)* or *foreign (outside The US)* market, and according to the linguistic tone surrounding the newspaper article.

To identify the linguistic tone of the articles, MonkeyLearn’s sentiment analysis automated process of understanding and sorting text data has been used. Apart from the machine-learning algorithm, MonkeyLearn’s sentiment analysis in its core is based on the Loughran and McDonald (2011) [20] dictionary (LM) and Harvard-IV-4 word list. Of the total number of articles used, articles bearing negative linguistic tone are 85.7%, and the rest are articles of net positive linguistic tone.

Next, to examine whether reported corporate misconduct article announcements have an impact on firms’ CARs, the study exerts the value-weighted market model CARs as calculated by the *Refinitiv Datastream Event Study Tool*.

Lastly, data on financial penalties assigned to the firms around each reported corporate misconduct is collected from the official US Data.gov website, and data on firm fundamentals for the period of observation, January 1995 –January 2020, is collected from *Refinitiv Datastream* (total assets, long- and short-term financial debt, total revenues, and pretax income from foreign operations).

## Methodology

The event study method is used to evaluate the economic impact of the reported corporate misconduct events published in the newspaper articles through the one-factor value-weighted market model (Carberry et al., 2018 [4]; Kaplansky & Levy, 2010 a, b [8, 9]). Analyses begin by computing the cumulative abnormal returns (CARs) around the reported corporate misconduct events considered. The abnormal returns (ARs) are defined as the difference between the actual rate of return of the stock considered and its ex-post expected rate of return over the whole length of the designated event window (MacKinlay, 1997) [21]. The Gujarati (1970) [22] technique is used to uncover the shifts in the market model estimate, setting 100 days in the estimation period ending 3 days prior to the designated event windows surrounding the event day, i.e. day 0. The event study estimates would be biased due to overlapping event windows if all published articles were considered as independent, thus a selection criterion called “the first announcement” is used. The selection criterion selects the articles, i.e. events, in a chronological sequence, where it starts with the first event in the sample, ignores all events showing up in the following 2 or 5 days-depending on the length of the event window [-5, +5], [-1, +1], and [0, +5]. In addition, the selection criterion continues with the second event ignores the events in the proposed range, and so on. This procedure results in 1,269 non-overlapping events (see Bernard, 1987) [23].

To observe the statistical impact of the reported corporate misconduct events on the CARs the estimations evolve around the following model (e.g., Berlinger et al., 2022 [19]; Ichev, 2018 [24]; Wagner et al., 2018 [18]; Donadelli et al., 2016b [11]; Kamstra et al., 2003 [25]; Brown & Warner, 1985 [26]):

$${CAR}_{i,t}=\gamma_{0}+{\gamma \prime }_{1}{CM}_{i,t}+\gamma_{2}{Level}_{i,t}+\gamma_{3}{Location}_{i,t}+\gamma_{4}{LT}_{i,t}+\gamma_{5}{Penalty}_{i,t}+{\gamma \prime }_{6}Z_{i,t}+\varphi_{t}+\pi_{t}+\epsilon_{t}$$ (1)

Where *CAR*_*i*,*t*_ is the cumulative abnormal return of firm *i* on event window *t*, *γ*_0_ is the regression intercept, *CM*_*i*,*t*_ is the type of reported corporate misconduct undertaken by a specific firm, and *Level*_*i*,*t*_ is a dummy variable denoting reported corporate misconducts done on a *corporation* level, and by *C-level* individuals otherwise. *Location*_*i*,*t*_ is a dummy variable equaling 1 if reported corporate misconducts take place in the home market and 0, otherwise. *Penalty*_*i*,*t*_ is the reported relative amount of USD fine around each reported corporate misconduct, and *LT*_*i*,*t*_ is a set of dummy variables indicating the linguistic tone used in a specific article. Lastly, *Z*_*ij*,*t*_ is a set of baseline controls (“size”- the natural logarithm of total assets, “leverage”–long- and short- term financial debt scaled by total assets, “revenue growth”–growth in revenues from the previous period, and “foreign exposure”–pretax income from foreign operations scaled by total assets). *φ*_*t*_ and *π*_*t*_ denote the industry and year controls.

## Results

### Event study

**Fig 1:**
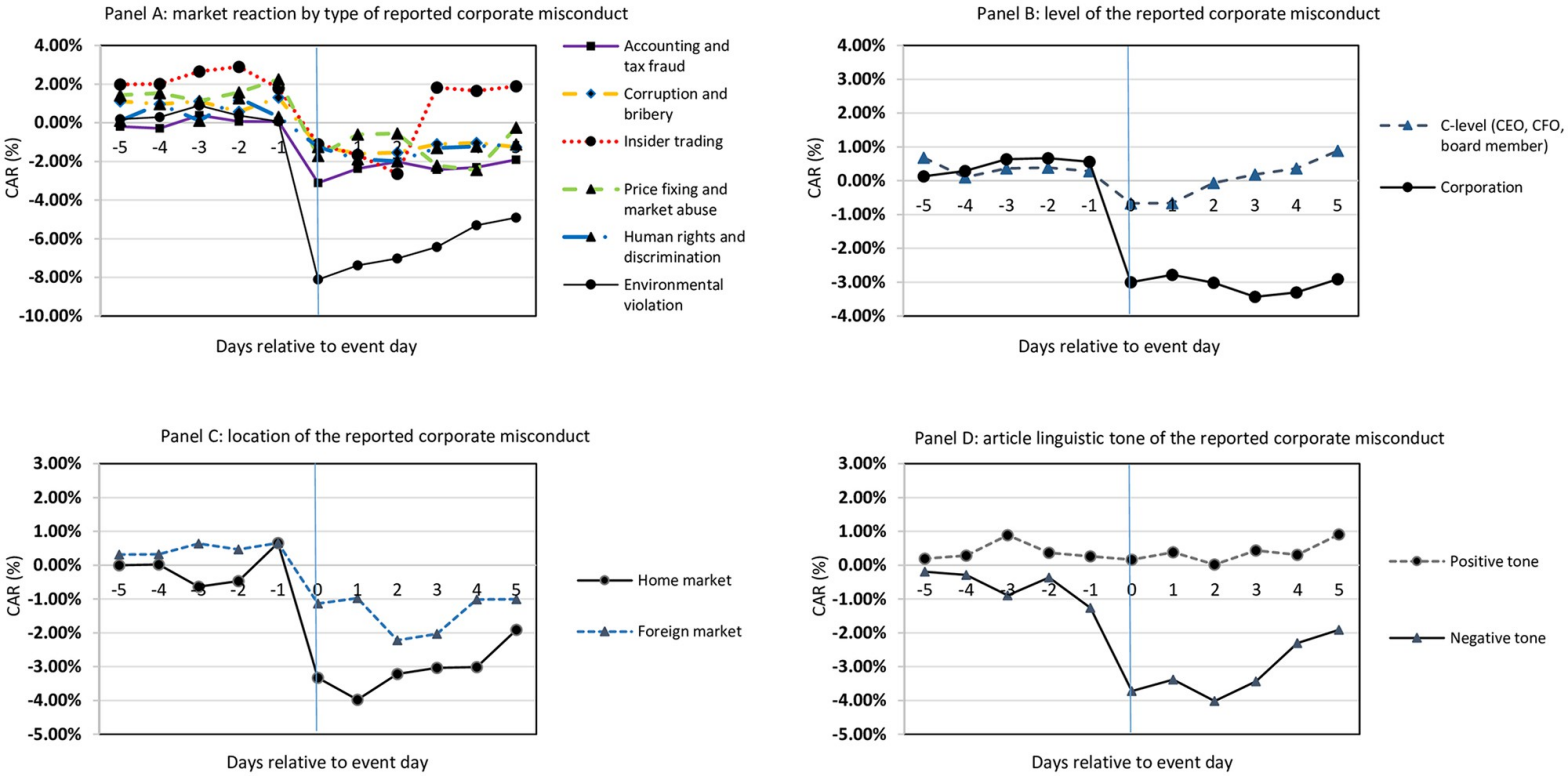
Market reaction around the reported corporate misconduct events. ***Notes*:** Panel A, panel B, panel C, and panel D of Fig 1 graphically show the results presented in Table 1.

Table 1 along with Fig 1 summarize the CARs around the reported corporate misconduct events. Panel A shows that the CARs for *all* types of reported corporate misconducts are negative and statistically significant at 1% level. Although negative and statistically significant already 5 days prior the event day, CAR estimates’ magnitude is more pronounced in the period after the initial newspaper announcement, hence once the information reaches broader audience. This CAR, for the [0, +5] window, is negative 4.104% with Patell Z score of -4.619. Lange & Washburn (2012) [27] define a reported corporate misconduct as a behaviour showing lack of socio-economic due concern for communities or the environment. This lack of due concern causes punitive actions from investors and stakeholders, translating into negative CARs for the firms (Grappi et al. 2013a [28]; Balabanis, 2013 [29]).

**Table 1:** Market reaction around the reported corporate misconduct events.

**Panel A. Type of reported corporate misconduct**					
**Event Window**	**No. of obs.**	**CAR (%)**	**Patell Z score**	**StdCsect Z**	**G.Sign Test Z**
*All reported misconducts*					
[-5, +5]	1,269	-2.747***	-3.033	-4.895	-2.691
[-1, +1]	1,269	-3.112***	-3.239	-5.129	-3.131
[0, +5]	1,269	-4.104***	-4.619	-7.228	-4.414
*Accounting and tax fraud*					
[-5, +5]	183	-3.112***	-2.554	-8.153	-4.725
[-1, +1]	183	-5.103***	-3.702	-9.100	-7.005
[0, +5]	183	-8.320***	-5.100	-12.203	-10.668
*Corruption and bribery*					
[-5, +5]	138	-1.205**	-2.441	-3.189	-2.500
[-1, +1]	138	-1.208**	-2.459	-3.192	-2.519
[0, +5]	138	-1.960***	-4.902	-5.157	-4.668
*Insider trading*					
[-5, +5]	38	-1.100**	-1.992	-2.551	-1.988
[-1, +1]	38	-1.115**	-1.960	-2.614	-2.462
[0, +5]	38	-1.528***	-2.944	-3.287	-3.270
*Price fixing and market abuse*					
[-5, +5]	153	-1.700**	-1.981	-2.109	-1.966
[-1, +1]	153	-1.785**	-2.002	-2.142	-2.088
[0, +5]	153	-2.114***	-2.541	-3.544	-2.125
*Human rights and discrimination*					
[-5, +5]	269	-1.252**	-1.989	-2.115	-2.414
[-1, +1]	269	-1.000*	-1.745	-1.990	-2.017
[0, +5]	269	-1.488***	-2.010	-2.322	-2.630
*Environmental violation*					
[-5, +5]	488	-8.114***	-7.245	-11.254	-2.554
[-1, +1]	488	-8.458***	-7.566	-11.741	-2.700
[0, +5]	488	-9.214***	-10.218	-16.855	-3.121
**Panel B. Level of the reported corporate misconduct**					
*Corporation*					
[-5, +5]	1,215	-2.002***	-2.623	-2.445	-1.568
[-1, +1]	1,215	-3.087***	-2.854	-2.886	-2.115
[0, +5]	1,215	-3.968***	-3.501	-3.521	-2.248
*C-level (CEO*, *CFO*, *board member)*					
[-5, +5]	54	-0.668	-1.593	-2.014	-1.235
[-1, +1]	54	-1.025**	-1.968	-2.144	-1.964
[0, +5]	54	-1.029**	-1.975	-2.621	-2.017
**Panel C. Location of the reported corporate misconduct**					
*Home market*					
[-5, +5]	1,015	-3.329***	-2.577	-1.998	-1.886
[-1, +1]	1,015	-3.410***	-2.309	-2.121	-2.062
[0, +5]	1,015	-4.423***	-2.800	-2.378	-2.380
*Foreign market*					
[-5, +5]	254	-1.136*	-1.835	-0.728	-0.835
[-1, +1]	254	-1.273**	-2.135	-1.415	-0.592
[0, +5]	254	-1.539**	-2.497	-2.294	-1.814
**Panel D. Article linguistic tone of the reported corporate misconduct**					
*Negative tone*					
[-5, +5]	1,088	-3.721***	-3.217	-2.858	-2.223
[-1, +1]	1,088	-5.500***	-3.385	-3.014	-2.874
[0, +5]	1,088	-8.218***	-5.476	-4.256	-3.267
*Positive tone*					
[-5, +5]	181	0.162	1.512	1.125	1.005
[-1, +1]	181	0.077	1.623	1.475	1.129
[0, +5]	181	0.568*	1.841	1.920	1.223

***Note*:** The Cumulative abnormal returns (CARs) are calculated using the value-weighted market model. 100 trading days are positioned in the estimation of the market model ending 3 days prior to the event window surrounding the event day, i.e. day 0. Only reported corporate misconduct events with non-overlapping event windows are used. The following procedure is used to select the events. The selection criterion, which has a label “the first occurrence”, selects events in chronological order (sequence). It starts with the first event in the sample, ignores all events showing up in the following 2 or 5 days–depending on the length of the event window ([-5, +5], [-1, +1] and [0, +5]). Next, it takes the second event ignores the events in the proposed range, and so on. Subsequent columns report the Patell Z-score, the standardized cross-sectional Z-score, and the generalized sign test, respectively. Asterisks *, **, and *** denote significance at 10%, 5%, and 1% levels, respectively.

While analysing different types of reported corporate misconducts, firms conducting *accounting and tax frauds* are found to experience negative CARs of about 8.320% for the [0, +5] window. This finding corresponds to Karpoff et al. (2008) [6] who find that firms pay substantial penalties for cooking the books, which in their case ranges from 8.8% and up to 24.5% in equity value loss. For the *corruption and bribery*, *insider trading*, and *price fixing and market abuse* reported corporate misconducts the event study estimates are as expected. Each event window yields negative and statistically significant CARs, indicating that firms get penalized in the financial markets for illegal activities and insider trading practices on both *corporation* and *C-level* (see Engelen, 2009 [5]). The CARs for the *human rights and discrimination* reported misconducts are also negative and significant, and in line with previous studies, such as Reichert et al. (1996) [30], and Rao & Hamilton (1996) [31]. Last but not least, largest by magnitude effects upon firms’ CARs are found for those firms engaging in *environmental valuation* reported misconducts. The CAR for the full event window, i.e. [-5, +5], is -8.114% statistically significant at 1%. Reported environmental misconducts can be sanctioned via explicit legal sanctions imposed through regulatory, civil, and criminal proceedings, and such proceedings infer significant reputational penalties and subsequently reduction in firm value (Preston, 2015) [32].

Panel B categorizes reported corporate misconducts by management level. Results show that firms are punished almost three times more when an act of misconduct is blamed on the *corporation* in general, rather than when specific individuals are pointed out in the media. When reported misconducts are done by *C-level* individuals, firms can simply solve the problem by firing these employees (Devers et al., 2009) [33]. Paruchuri & Misangyi (2015) [34] find that, quite often, firms in attempt to divert negative media attention assign blame to specific C-level individuals so the negative sentiment fades away from the entire firm. These individuals would be perceived by the investors as “bad apples” hence the bad media attention would be isolated to an individual level (Connelly et al., 2016) [35].

When an act of reported misconduct is blamed on a corporation level, this signals to structural problems within the specific firm, which in turn translates to more (by magnitude) negative CARs (Shadnam & Lawrence, 2011) [36]. Collective inclusion in a reported misconduct makes more damage on organizations than individual wrongdoing, because the more people are involved in a reported misconduct action, the more it can accomplish (Palmer, 2012) [37].

The estimates in panel C show that reported corporate misconducts taking place in the home market result in more negative CARs in comparison to the reported misconducts done in the foreign markets. Looking at the [0, +5] window, the home market CAR is -4.423% statistically significant at 1%, while the CAR for the foreign market reported misconducts is just -1.539%, statistically significant at 5%. One potential explanation for this result goes along Barberis & Thaler (2003) [38], who claim that investors exhibit a “home country” bias, i.e., they focus on investing in firms headquartered in their own countries since they are more familiar with the domestic market. Another explanation evolves around the geographic proximity between specific events and the financial markets. Engelberg & Parsons (2011) [16] observe media effects on stock returns at a local level. They find that local press coverage stimulates local trading activity, and increases the trading volume of local investors up to 50%.

Panel D analyzes the linguistic tone of the newspaper articles written on the reported corporate misconducts. The results show evidence that the negative linguistic tone in the media negatively affects firms’ stock market performance, inferring negative and statistically significant CARs for all event windows. For the articles bearing positive linguistic tone, no strong statistical evidence is found, which does not necessarily mean that there is no underlying effect upon these CARs but that cannot simply be observed, possibly due to the small sample size. These findings are consistent with previous research findings in the finance literature pointing to the fact that stock market reaction to news bearing specific linguistic tone tends to be large, significant, and asymmetric (e.g., Ranco et al., 2015 [39]; Brans & Scholtens, 2020 [40]).

### Regression analyses

Table 2 presents results from estimating the effect of reported corporate misconducts on firms’ CARs. Columns (1) to (6) summarize the results of the regression analysis in model (1) exercised upon the [-5, +5], [-1, +1], and [0, +5] CAR event windows. Robust standard errors, industry-, and year- controls are used to support the rigidity of the estimations.

**Table 2:** The impact of the reported corporate misconduct events on the CARs.

Dependent variable	Value-weighted MM CARs for all firms					
	(1)	(2)	(3)	(4)	(5)	(6)
	[-5, +5]	[-5, +5]	[-1, +1]	[-1, +1]	[0, +5]	[0, +5]
Accounting and tax fraud	-0.0321*** **(-3.12)**	-0.0318*** **(-3.00)**	-0.0488*** **(-3.87)**	-0.0479*** **(-3.62)**	-0.0793*** **(-4.22)**	-0.0774*** **(-4.11)**
Corruption and bribery	-0.0113 (-1.56)	-0.0110 (-1.51)	-0.0119 (-1.62)	-0.0115 (-1.55)	-0.0198* **(-1.89)**	-0.0188* **(-1.79)**
Insider trading	-0.0102** **(-2.15)**	-0.0115** **(-2.21)**	-0.0100** **(-2.00)**	-0.0106** **(-2.01)**	-0.0163*** **(-2.74)**	-0.0159*** **(-2.63)**
Price fixing and market abuse	-0.0174** **(-1.97)**	-0.0172** **(-1.96)**	-0.0177** **(-1.99)**	-0.0168** **(-1.97)**	-0.0208*** **(-2.88)**	-0.0202*** **(-2.74)**
Human rights and discrimination	-0.0127** **(-2.05)**	-0.0122** **(-1.99)**	-0.0102* **(-1.66)**	-0.0100* **(-1.64)**	-0.0158*** **(-2.56)**	-0.0152*** **(-2.53)**
Environmental violation	-0.0914*** **(-12.52)**	-0.0909*** **(-12.20)**	0.1022*** **(-15.17)**	0.1019*** **(-15.02)**	0.1121*** **(-16.02)**	0.1100*** **(-14.12)**
Level	-0.0212*** (-2.88)	-0.0208*** (-2.67)	-0.0338*** **(-4.24)**	-0.0329*** **(-4.00)**	-0.0447*** **(-5.58)**	-0.0435*** **(-4.23)**
Location	-0.0315*** **(-3.22)**	-0.0310*** **(-3.00)**	-0.0340*** **(-3.61)**	-0.0335*** **(-3.58)**	-0.0402*** **(-4.39)**	-0.0400*** **(-4.38)**
Negative tone	-0.0385*** **(-5.11)**	-0.0374*** **(-5.02)**	-0.0412*** **(-5.78)**	-0.0410*** **(-5.76)**	-0.0458*** **(-5.99)**	-0.0451*** **(-5.84)**
Positive tone	0.0013 (0.37)	0.0009 (0.24)	0.0085 (1.52)	0.0080 (1.50)	0.0097 (1.63)	0.0095 (1.60)
Penalty	-0.0103* **(-1.88)**	-0.0100* **(-1.69)**	-0.0076 (-1.59)	-0.0070 (-1.50)	-0.0121** **(-1.99)**	-0.0118** **(-1.97)**
Size	0.0587*** **(4.01)**		0.0602*** **(4.74)**		0.0610*** **(4.82)**	
Leverage	-0.0132** **(-1.96)**		-0.0138** **(-1.99)**		-0.0135** **(-1.97)**	
Revenue growth	0.0078** **(2.15)**		0.0081** **(2.19)**		0.0084** **(2.23)**	
Foreign exposure	-0.0014** **(-2.10)**		-0.0025** **(-2.23)**		-0.0031** **(-2.28)**	
# of obs.	1,269	1,269	1,269	1,269	1,269	1,269
R-squared	0.337	0.291	0.336	0.297	0.386	0.355
Robust st. errors	Yes	Yes	Yes	Yes	Yes	Yes
Industry controls	Yes	Yes	Yes	Yes	Yes	Yes
Year controls	Yes	Yes	Yes	Yes	Yes	Yes

***Note*:** This table summarizes the results of the regression analysis in model (1). CARs are calculated using the value-weighted market model for the [-5, +5], [-1, +1], and [0, +5] windows. The regressions focus on the impact of the reported corporate misconduct announcements upon firm CARs. Firm fundamentals are used as controls. T-statistics are in parenthesis, and asterisks *, **, and *** denote significance at 10%, 5%, and 1% levels, respectively.

Observing the estimates for the [-5, +5] window, both models, with- and without control variables, show results corresponding to the findings of the event study assessment in the previous section. Namely, each type of reported corporate misconduct negatively and statistically significantly affects the CARs, apart from *corruption and bribery* for which the statistical significance is less than 10%. Analyzing the source of reported misconduct within the firms from a management level perspective, shows that reported misconducts blamed on a corporation level yield about -2.1% towards the CAR of the firms. This finding corresponds to Connelly et al. (2016) [35] who find that corporations’ returns suffer much more when there is no individual within the firm to take the blame for the reported misconduct made. Furthermore, reported misconducts occurring in the home market produce more negative CARs than reported misconducts taking place in foreign markets. Ichev & Marinč (2018) [24] explain that the geographic proximity between specific events and the financial markets plays important role for firms’ CARs, especially when the events take place in the home market.

Next, articles written in a negative linguistic tone are found to negatively and significantly affect firms’ CARs. Positive linguistic tone articles positively affect the CARs, but no statistical significance is recorded, yet again. This finding is consistent with Conrad et al. (2002) [41], who find that stock price response to negative news is larger than to positive news, and depends on the rise of the relative level of the market. Observing the financial penalty in terms of USD assigned to each firm engaging in a reported corporate misconduct, a negative and statistically significant at 10% impact towards firms’ CARs is recorded as well. According to Berlinger et al. (2022) [19], this result has two important implications: i) investors reaction to the financial penalty information associated with the reported misconduct would be even stronger towards firms’ CARs, and ii) having the information about the penalty imposed publicly available is an indicator itself of the freedom of speech in the country. The controls for firm size, leverage, revenue growth, and firm foreign exposure are as expected: larger firms contribute to higher CARs, which is evident from the fact that all firms in our sample are NYSE and NASDAQ listed firms; highly leveraged firms are affected more negatively than less leveraged firms; and firms with higher revenue growth show better performance than firms with lower revenue growth in terms of CARs. Finally, firms with foreign exposure are not positively perceived by the stock market resulting in negative CARs.

Regression coefficients presented in the other two event windows, i.e. [-1, +1], and [0, +5], draw similar conclusions. There is a negative impact from the reported corporate misconducts towards firms’ CARs recorded, which is stronger for the reported misconducts taking place in the home market as well as for the reported misconducts blamed on the level of the corporation. Linguistic tone of the media articles is skewed towards the negative tone in terms of impact upon the CARs, and the control variables are as expected. It is important to note that the magnitude of the estimates for the [0, +5] window is somewhat higher compared to the other two windows, suggesting that the market participants react more to the information once the information reaches broader audience. As it goes for the financial penalties control, the coefficients are statistically insignificant for the [-1, +1] window which explains the fact that for the bigger part of the reported corporate misconducts penalties are not yet determined in this short time window.

## Conclusion

Examining and understanding the relationship between the media, the stock markets, and the socio-economic environment has become more critical as stock markets become more important societal arbiters of ethical behavior (Barberis & Thaler, 2003) [38].

This study examines media reported corporate misconducts and documents a negative impact of *all* types of reported corporate misconducts upon the cumulative abnormal returns for the firms listed on the US financial markets. Tests reveal that the impact of the reported misconducts upon firms’ CARs differ among the levels of management within the firm, market location of the reported misconducts, and the linguistic tone of the newspaper article(s) covering the specific reported misconduct. Additional tests show that financial penalties imposed, size, leverage, revenue growth, and the degree of firms’ foreign exposure play a significant role in companies’ financial performance during the period of observation. The results documented imply that firms’ weaker stock market performance comes as a result of negative media coverage, unethical behavior conducted in the home market, when firms are blamed for environmental violation, and when the blame of engaging in unethical behavior is absorbed by the firm as an entity rather than by a specific individual within the firm.

This study has some limitations too. It is consciously limited to the use of newspapers as a credible mass media source to create the sample of reported corporate misconducts. Another limitation is that the analyses do not differentiate among various types of investors. Yet another limitation could be that firms’ ESG scores are not incorporated in the analyses. Nevertheless, it is important and worth documenting these effects as the firms may find the bottom-line results as an incentive to re-consider their existing actions and strategies.

## Supporting information

S1 Dataset Package of all datasets. (ZIP)
