# Supplementary material for: Reported corporate misconducts: The impact on the financial markets
Source: PLoS One. 2023 Feb 9;18(2):e0276637. doi: 10.1371/journal.pone.0276637 (PMC9910724; doi:10.1371/journal.pone.0276637)
Supplement: S1 Dataset — (ZIP) [file pone.0276637.s001.zip › S2 Code Keywords.pdf]

ProQuest article search engine code keywords

1. Type of corporate misconduct

1.1. Accouting fraud
1.2. Tax fraud
1.3. Other fraud
1.4. Corruption and bribery
1.5. Insider trading
1.6. Price fixing and market abuse
1.7. Human rights
1.8. Discrimination
1.9. Miscellaneous
1.10. Environmental violation
1.11. Sexual harassment

2. Level of corporate misconduct

2.1. Top (CEO, CFO, top management, board members)
2.2. Other employees
2.3. Corporation
2.4. Unknown, unclear and others

3. Location of corporate misconduct

4.1. Transgression in home market
4.2. Transgression in foreign market

4. Media coverage of corporate misconduct

7.1. Number of newspapers covering incident
7.2. Total number of words in articles
7.3. Domestic coverage or international coverage
